# Loeffler endocarditis revealing chronic eosinophilic leukaemia with FIP1L1-PDGFRA rearrangement: a case report

**DOI:** 10.1093/ehjcr/ytaf218

**Published:** 2025-05-02

**Authors:** Raid Faraj, Zineb El Bougrini, Aatif Benyass, Youssef Sekkach, Ilyasse Asfalou

**Affiliations:** Cardiology Center, Mohammed V Military Instruction Hospital of Rabat, Mohammed V University of Rabat, 10000 Rabat, Morocco; Department of Internal Medicine, Cardiology Center, Mohammed V Military Instruction Hospital of Rabat, Mohammed V University of Rabat, 10000 Rabat, Morocco; Cardiology Center, Mohammed V Military Instruction Hospital of Rabat, Mohammed V University of Rabat, 10000 Rabat, Morocco; Department of Internal Medicine, Cardiology Center, Mohammed V Military Instruction Hospital of Rabat, Mohammed V University of Rabat, 10000 Rabat, Morocco; Department of Internal Medicine, Cardiology Center, Mohammed V Military Instruction Hospital of Rabat, Mohammed V University of Rabat, 10000 Rabat, Morocco

**Keywords:** Loeffler Endocarditis, Chronic eosinophilic leukaemia, FIP1L1-PDGFRA rearrangement, Case report

## Abstract

**Background:**

Hypereosinophilic syndrome (HES) is an infrequent multisystemic disorder with a serious prognosis, defined by persistent marked eosinophilia (>1500 eosinophils/mm3) associated with organ damage from eosinophil-mediated cytotoxicity. Cardiac involvement is a significant and unpredictable complication of hypereosinophilic syndrome, particularly prevalent in patients carrying the FIP1L1-PDGFRA fusion.

**Case summary:**

Reported is a case of chronic eosinophilic leukaemia (CEL) with a FIP1L1-PDGFRA rearrangement, diagnosed in a 31-year-old patient presenting with Loeffler endocarditis. Intracardiac thrombi and embolic cerebral infarctions complicated the case. The patient demonstrated haematological remission following chemotherapy, and anticoagulation treatment led to thrombi resolution.

**Discussion:**

This case highlights that Loeffler endocarditis can present as the primary and sole manifestation of chronic eosinophilic leukaemia. Effective collaboration between cardiologists and internists is crucial for timely diagnosis and comprehensive management, ultimately resulting in enhanced outcomes.

**Learning points**

- To be aware of Loeffler endocarditis as a rare and severe complication of chronic eosinophilic leukaemia.
- To be aware of the role of cardiac magnetic resonance imaging in the diagnosis of Loeffler endocarditis.

## Introduction

Myeloproliferative hypereosinophilic syndrome (HES) is an uncommon condition, and its actual prevalence remains uncertain. Within the United States, the estimated occurrence ranged from 0.36 to 6.3 cases per 100 000 individuals.^1^ While several subtypes of HES demonstrate an equitable distribution across genders, HES associated with genetic anomalies affecting the tyrosine kinase receptors platelet-derived growth factor receptor alpha (PDGFRA) and platelet-derived growth factor receptor beta [PDGFRB] exhibit a discernible male predominance. This predilection is especially evident among the male population aged between 20 and 50 years, encompassing both paediatric and adult individuals.^2^ Typical organs affected are the skin, lungs, and gastrointestinal tract. In rarer instances, there is a potential for cardiovascular and neurological complications to arise, which could be life-threatening.

Only a limited number of reports regarding Loeffler endocarditis in patients with CEL exist, especially in Asia.^3^ To the best of our knowledge, this represents the first documented case of CEL featuring FIP1L1/PDGFRA rearrangement, combined with Loeffler endocarditis in Africa.

Our paper was written according to the CARE guidelines.^4^

## Summary figure

**Table:** 

*Baseline*	*The patient gradually presented pronounced weight loss, reduced appetite, along with episodes of intermittent fever. He also reported experiencing headaches and bilateral extremity paraesthesia*
*1 month later at 22:00*	*Patient presents to the emergency room with chest pain and dyspnoea*
*23:00*	*ECG as well as the echocardiography showed no abnormalities. The laboratory tests revealed an elevated troponin level as well as a significant hypereosinophilia.* *Cerebral CT scan revealed multiple small acute infarcts at the bi-hemispheric anterior junction*
Next day at 9:00 :	*Cardiac magnetic resonance imaging was performed, revealing the presence of endomyocardial fibrosis along the lateral wall of the left ventricle, accompanied by mural thrombi. Comprehensive examinations to elucidate underlying factors, encompassing bone marrow aspirate coupled with molecular investigations were performed.*
12:00	*After a multidisciplinary team discussion, the patient's treatment regimen was initiated, involving the administration of Imatinib and Hydroxyurea, coupled with curative anticoagulation.*
15 Days Later	*The Patient's Condition Is Stable, And A Subsequent Cardiac Mri Showed The Resolution Of The Thrombi. The Complete Blood Count Demonstrated A Resolution Of Hypereosinophilia.*

## Case presentation

We present a case study involving a 31-year-old patient who was admitted to the emergency department due to atypical chest pain and exertional dyspnoea. The patient had a familial history of early sudden deaths in two paternal uncles. The patient reported a gradual onset of pronounced weight loss, reduced appetite, and intermittent fever over the past month. Additionally, the patient experienced headaches and paraesthesia in both extremities.

**Figure 1:**
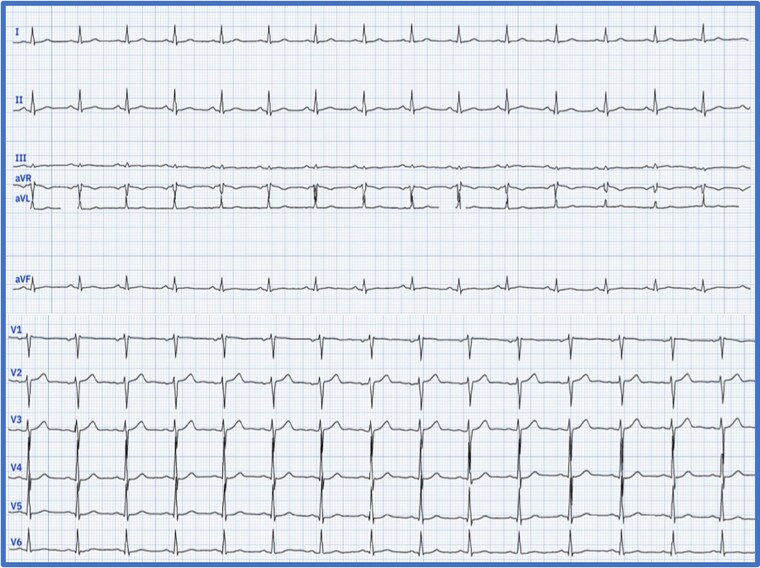
Patient’s ECG showing no abnormalities.

**Figure 2:**
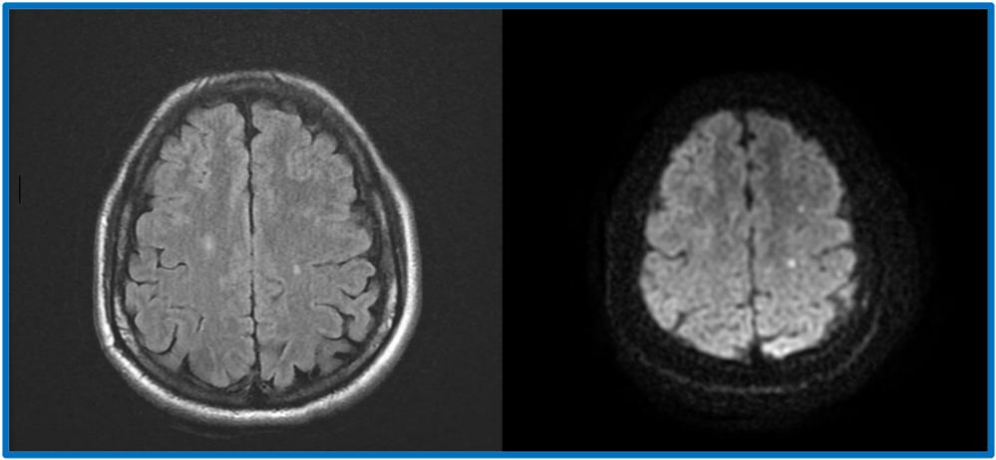
Patient’s cerebral MRI showing multiple small acute infarcts at the bi-hemispheric anterior junction.

**Figure 3:**
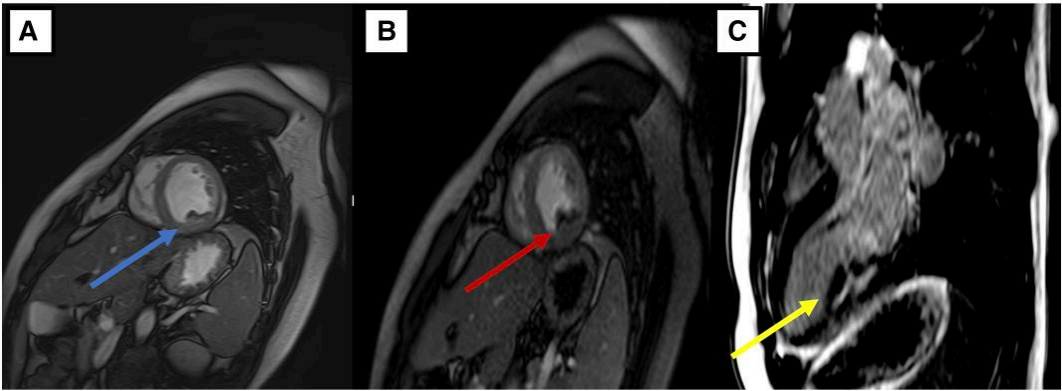
(*A*) Cine bSSFP short axis imaging showing a flat wall thrombus (blue arrow) lining the inferolateral wall (median and basal). (*B*) Rapid multi-slice myocardial perfusion imaging showing the presence of intracavitary masses adhering to the lateral (median) wall of the LV, not contrasting with the rest of the myocardium (red arrow). (*C*) late-gadolinium enhancement in three-chamber view characterizing the typical triple-layered appearance of endomyocardial fibrosis.

**Figure 4:**
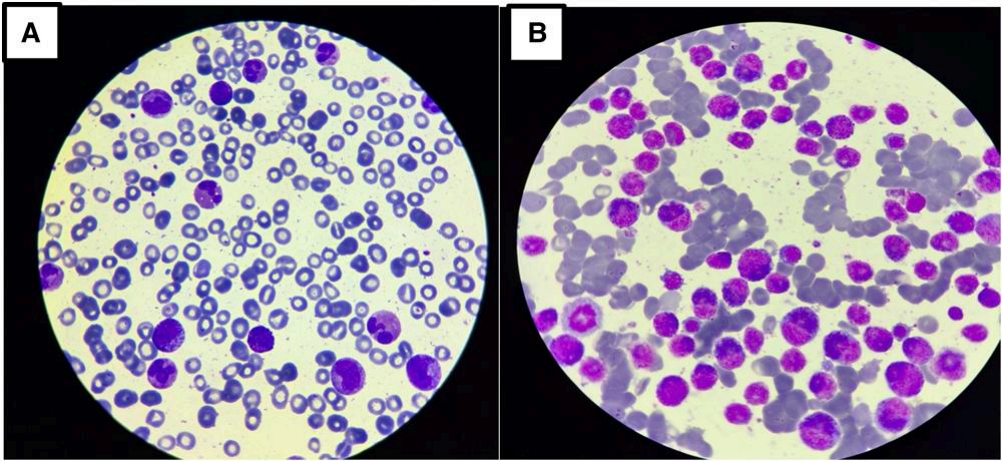
(*A*) blood smear (May–Grünwald–Giemsa stain, ×100 objective) showing numerous eosinophilic polymorphonuclear cells with a polysegmented nucleus, vacuolated cytoplasm, and numerous precursors of the myeloid lineage. (*B*) Bone marrow smear (MGG stain, ×100) showing numerous precursors of the dystrophic eosinophilic lineage containing large basophilic granules.

Upon presentation, the patient exhibited stable haemodynamic and respiratory status, with no remarkable findings during the clinical examination. On admission, the vital signs were as follows: heart rate of 85 beats per minute, blood pressure of 120/80 mmHg, temperature of 37.5 °C, respiratory rate of 18 breaths per minute, and oxygen saturation of 98% on room air. The clinical examination revealed no remarkable findings. Electrocardiogram (ECG) (*Figure 1*) was performed and yielded normal results with no detectable abnormalities. Laboratory tests revealed leukocytosis [36.2 × 10³/μL (reference range, ∼4.5–11.0 × 10³/μL)] with marked eosinophilia [57.9%; 21 × 10³/μL (normal absolute eosinophil count, <0.5 × 10³/μL)], non-regenerative anaemia [8 g/dL (normal range, ∼13.5–17.5 g/dL)], and thrombocytopenia [70 × 10³/μL (normal platelet count, 150–400 × 10³/μL)]. Vitamin B12 levels were elevated [>6000 pg/mL (normal, 200–800 pg/mL)], as well as Immunoglobulin E [991 IU/mL (normal <100 IU/mL in adults)]. Additionally, there was an elevated troponin level of 2477 ng/L, (normal <0.04 ng/mL), which subsequently increased to 3531 ng/L. Transthoracic echocardiogram (TTE) did not show signs of restrictive cardiomyopathy and was unable to confirm the presence of diffuse or localized endocardial thickening. On the following day, a cardiac MRI revealed a mural thrombus along the basal and medial segments of the inferolateral wall of the left ventricle (LV), with no evidence of myocarditis. Both the LV and right ventricle (RV) exhibited normal function. Cine bSSFP short-axis imaging showed a flat thrombus lining the inferolateral wall, while rapid multi-slice myocardial perfusion imaging identified intracavitary masses adhering to the mid-lateral LV wall, which did not enhance with contrast. Late gadolinium enhancement in a three-chamber view demonstrated the characteristic triple-layered pattern, confirming endomyocardial fibrosis (*Figure 2*). A cerebral MRI revealed multiple small acute infarcts at the bi-hemispheric anterior junction (*Figure 3*). The blood smear showed numerous eosinophilic polymorphonuclear cells with a segmented nucleus and vacuolated cytoplasm, along with many precursors of the myeloid lineage (*Figure 4-A*). Meanwhile, the bone marrow aspirate revealed a significant number of dystrophic eosinophilic lineage precursors containing large basophilic granules (*Figure 4-B*).

After a multidisciplinary team discussion, the patient's treatment plan included prednisone at a dose of 1 mg/Kg/day, Imatinib 100 mg daily, Hydroxyurea, and Warfarin. The use of warfarin was guided by the presence of intracardiac thrombi and the prothrombotic state associated with eosinophilic myocarditis. Subsequent to these interventions, a diagnosis of CEL with FIP1L1-PDGFRA rearrangement was supported by the results of FISH analysis.

## Follow-up

**Figure 5:**
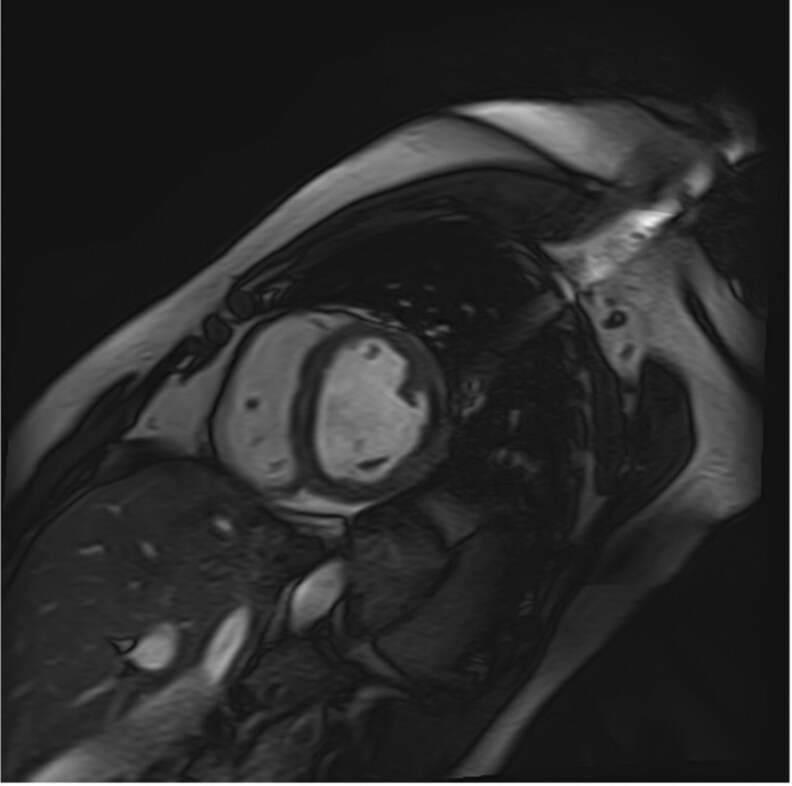
A follow-up cardiac MRI displayed a complete disappearance of the mural thrombus.

After 15 days of treatment, the patient exhibited significant clinical improvement. A follow-up cardiac MRI demonstrated complete resolution of the previously observed thrombi (*Figure 5*), and a complete blood count showed a marked reduction in hypereosinophilia. Notably, while a previously reported Korean case^3^ achieved full recovery with low-dose imatinib alone, our patient’s treatment course was more complex. Despite the improvement in thrombus resolution and eosinophilic counts following chemotherapy and anticoagulation, the patient developed a cytopenic state attributed to imatinib therapy. This adverse effect necessitated a dose reduction of imatinib, prompting a switch to a bi-day dosing regimen. This case thus highlights the critical need for careful dose adjustment when managing CEL with FIP1L1-PDGFRA rearrangement, especially in balancing the efficacy of imatinib in controlling the leukemic process against the risk of haematologic toxicity. The clinical challenge lay in simultaneously achieving thrombus resolution and managing the cytopenic effects of imatinib, underscoring the importance of vigilant monitoring and individualized treatment modifications.

## Discussion

CEL is a haematological rarity distinguished by the clonal expansion of eosinophilic lineage cells in the peripheral circulation, bone marrow, and various anatomical sites.^5^ The convergence of Loeffler endocarditis with CEL, particularly when associated with the FIP1L1-PDGFRA rearrangement, poses intricate challenges in both diagnosis and management. The underlying pathophysiology involves a complex interplay of molecular events. The FIP1L1-PDGFRA fusion gene results in a constitutively activated tyrosine kinase, leading to uncontrolled proliferation and activation of eosinophils.^6^ This enhanced eosinophilic activity, characterized by the release of cytotoxic granule proteins, contributes to endomyocardial fibrosis, a hallmark of Loeffler endocarditis.^7^ The intricate molecular landscape underscores the systemic nature of eosinophilic disorders and their propensity to involve multiple organ systems, particularly the cardiovascular system.^8^ CEL manifests with elevated serum vitamin B12 levels, chromosomal aberrations, anaemia, and/or thrombocytopenia. Additionally, there are increased levels of serum tryptase, hepatomegaly, splenomegaly, and the presence of circulating leukocyte precursors.^2^ Patients with Loeffler endocarditis may present with weight loss, fever, cough, rash, and symptoms related to congestive heart failure. In the 33 cases of Loeffler's endocarditis associated with a mural thrombus that was reported in the literature, the predominant symptoms were dyspnoea and fever, present in 63% and 30% of the patients, respectively, whereas thoracic pain, which was the chief complaint in our patient, was reported in only 15% of cases.^9^ Distinguishing Loeffler endocarditis, from constrictive pericarditis, can be challenging. Physical indicators in constrictive pericarditis that aid in discerning between the two conditions include the presence of a pericardial knock, the typically nonpalpable apex, and the absence of regurgitation murmurs in most cases. Only five patients with CEL and FIP1L1/PDGFRA rearrangement were reported in the literature^3,10^ but only one associated this condition with Loeffler endocarditis. In contrast to our patient, in whom cardiac MRI played a pivotal role in confirming the diagnosis and monitoring Loeffler endocarditis, the case reported in Korea relied on transthoracic echocardiography (TTE). The unique capability of cardiac MRI to reveal subendocardial late gadolinium enhancement (LGE) provides an early insight into endocardial fibrosis before functional consequences become apparent. In the context of HES patients, this imaging modality reduces the imperative for invasive biopsies. Notably, on cardiac MRI, the recognition of endocardial fibrosis in conjunction with a left ventricular apical thrombus is visually marked by a non-enhancing hypointensity situated between the enhancing endocardium and the bright blood pool, creating a discernible ‘sandwich appearance’^11^ The disease usually has a slow onset, progressing to increasing degrees of right and left heart failure, and the overall prognosis for individuals with Loeffler endocarditis is generally poor. The severity of the prognosis depends on the location of involvement in the heart.^7^ The prognostic outcome in Loeffler endocarditis is closely tied to the successful management and control of eosinophilia.^12^ The introduction of targeted therapies, notably tyrosine kinase inhibitors like imatinib, has significantly altered the treatment paradigm for CEL with FIP1L1-PDGFRA rearrangement. These agents, through selective inhibition of abnormal tyrosine kinase activity, have demonstrated effectiveness in regulating eosinophilic proliferation and improving cardiac manifestations.^6^ In the case of our patient, both haematologic abnormalities and cardiac involvement were entirely resolved with imatinib and anticoagulation, despite the need to reduce Imatinib doses due to a cytopenic state. Nevertheless, achieving a nuanced equilibrium between addressing haematologic irregularities and managing cardiac complications demands a patient-specific strategy.

A key lesson from this case is the potential benefit of earlier detection of CEL through systematic eosinophilia screening in patients presenting with unexplained embolic or cardiac events. Given that CEL is rare and its initial manifestations may be subtle, a routine evaluation—including a complete blood count with differential—in patients with unexplained embolic phenomena or cardiac dysfunction could facilitate earlier diagnosis. When an elevated eosinophil count is identified, further workup, including molecular studies for clonal eosinophilia (e.g. testing for the FIP1L1-PDGFRA rearrangement), should be pursued to confirm the diagnosis. In addition, incorporating advanced imaging modalities such as cardiac MRI into the diagnostic algorithm can help detect early cardiac involvement—specifically, subtle endocardial fibrosis evidenced by LGE—even before overt clinical symptoms develop. Early identification of these changes could prompt the timely initiation of targeted therapies, such as imatinib, potentially arresting the progression of endomyocardial fibrosis and mitigating thromboembolic complications. This proactive approach not only enhances patient outcomes by preventing irreversible cardiac damage but also offers a valuable strategy for the management of similar patients in the future.

## Conclusion

The convergence of Loeffler endocarditis with CEL, particularly in the context of FIP1L1-PDGFRA rearrangement, underscores the complexity of eosinophilic disorders with cardiovascular involvement. The ongoing collaboration between cardiologists and haematologists, coupled with ongoing research efforts, holds promise for gaining a deeper understanding of the complex pathogenesis and advancing therapeutic strategies in this rare and challenging disease.

## Data Availability

No supplementary material is available.

## Lead author biography

I am a Moroccan cardiology fellow with a strong interest in cardiac imaging and interventional cardiology. Currently pursuing my fellowship, I am committed to integrating diagnostic accuracy with advanced therapeutic strategies to improve cardiovascular care. My research has been presented at international conferences, including the ESC Congress, and published in reputable cardiology journals.
